# Plasmon-Activated Water can Prolong Existing Sea-Ice Habitats to Potentially Save Polar Bears

**DOI:** 10.1038/s41598-019-46867-5

**Published:** 2019-07-18

**Authors:** Chih-Ping Yang, Yi-Hao Wu, Hui-Yen Tsai, Jen-Chang Yang, Yu-Chuan Liu

**Affiliations:** 1 Department of Biochemistry and Molecular Cell Biology, School of Medicine, College of Medicine, Taipei Medical University, No. 250, Wuxing St., Taipei, 11031 Taiwan; 2 Graduate Institute of Nanomedicine and Medical Engineering, College of Biomedical Engineering, Taipei Medical University, No. 250, Wuxing St., Taipei, 11031 Taiwan; 3 Cell Physiology and Molecular Image Research Center, Wan Fang Hospital, Taipei Medical University, Taipei, Taiwan

**Keywords:** Applied physics, Environmental impact, Structural properties

## Abstract

Due to increasing global warming resulting from the greenhouse effect, subsequent environmental impacts and corresponding ecological influences are unavoidable. These problems are becoming more serious with time. Due to rising temperatures, the survival crisis of polar bears is a very often reported issue, because polar bears are encountering shortened seasons for catching prey on their sea-ice habitat. In this work, we report an innovative and facile strategy to save polar bears via prolonging the existence of ice layers based on plasmon-activated water (PAW). PAW with a reduced hydrogen-bonded network can be created by letting bulk deionized (DI) water flow through supported gold nanoparticles (AuNPs) under resonant illumination. Experimental results indicated that the freezing time of PAW was faster than that of DI water. In contrast, the melting time of frozen PAW was slower than that of the frozen DI water. Because the PAW with reduced hydrogen bonds (HBs) is in a high-energy state, it can more easily transform into a stronger HB structure in a low-energy state during cooling when freezing. This is accompanied by the release of more available energy, resulting in more-perfect tetrahedral symmetrical ice. Similar results were observed for solutions with 3 wt% NaCl, which is similar to the salinity of sea water. Moreover, the heat required to melt frozen PAW was ca. 7.6% higher than that of frozen DI water. These interesting phenomena suggest that prolonging the existence of solid ice can be achieved in a PAW-based system. Moreover, a system of AuNP-coated filter paper in DI water or in a DI water solution (3 wt% NaCl) under resonant illumination could work to prolong the presence of solid ice, compared to a system of AuNP-free filter paper. This innovative concept has emerged as a practical strategy to save polar bears and for other related applications.

## Introduction

Since the continued increase in global temperatures is apparently irreversible, causing serious reductions in the sea-ice habitats of polar bears, a strategy to prolong existing sea-ice habitats based on treated water may provide an alternative and practical way forward. Compared to other solvents, water possesses many unique properties due to its flexible dynamic hydrogen-bonded network. Hydrogen bonds (HBs) are broken and reformed at picosecond-scale equilibrium^1–5^. Because a molecular-level understanding of water’s structure is still unavailable, the commonly described chemical and physical properties of water are related to inert bulk water with strong HBs. Actually, water properties markedly differ from those of normal bulk water when it is in a confined environment^6,7^ or in contact with hydrophobic surfaces^8,9^. However, these interesting properties of water only exist in a confined environment or at the interfacial phase, limiting their applications.

In 2014, our group first created active and stable liquid water innovatively prepared using resonantly illuminated gold nanoparticles (AuNPs)^10^. The resulting plasmon-activated water (PAW) with reduced HBs exhibits distinct properties at room temperature, which significantly differ from the properties of untreated bulk deionized (DI) water. Because PAW can be innovatively applied in various fields, interesting PAW-related papers on green energy, medicine, chemical reactions, and physical processes have continuously been published by our group^11–21^. Compared to conventional DI water, the prepared PAW exhibits a lower specific heat and a corresponding lower boiling point^15^. It also possesses a higher vapor pressure and osmosis^10^. Moreover, the well-known Mpemba effect^22^ can be successfully explained by our proposed hypothesis based on the PAW-derived idea of water energy and HBs^15^. As shown in the literature, engineered water was also prepared using electro-spray technology to inactivate food-borne microorganisms^23,24^. In this work, we report interesting phenomena of treated PAW of it freezing faster and melting more slowly, which can prolong the existence of ice layers of both fresh and seawater.

## Results and Discussion

### Freezing and melting processes of PAW (and its NaCl solution) compared to DI water (and its NaCl solution)

**Figure 1:**
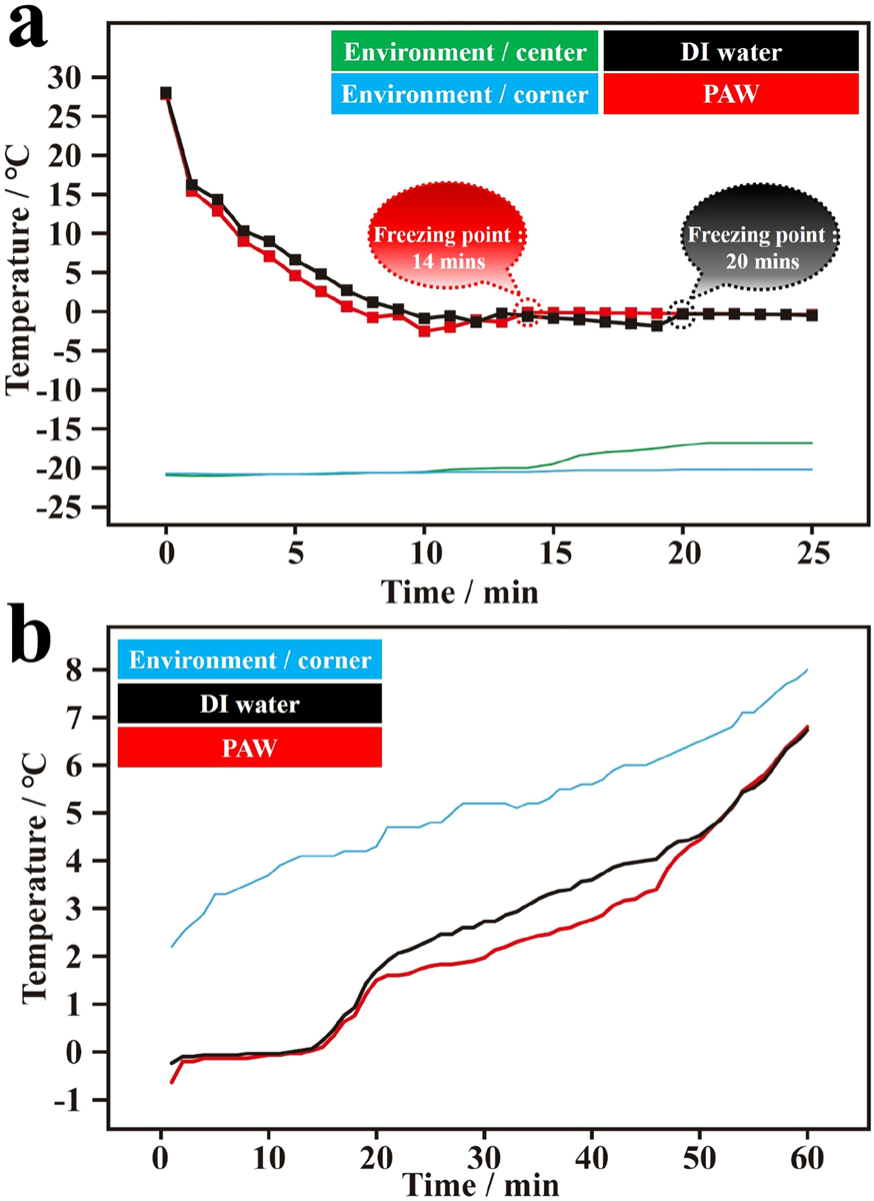
Temperature-time dependencies of plasmon-activated water (PAW) and deionized (DI) water during freezing and melting processes. (**a**) Temperature-freezing time dependencies of PAW (red line) and DI water (black line) in an ice bath of a salt-ice-water system (the temperature was controlled to ca. −20 °C). The green and blue lines represent the environmental temperatures measured at the center point and at a corner point, respectively, in the ice bath. (**b**) Temperature-melting time dependencies of frozen PAW (red line) and frozen DI water (black line) in a cold-water bath (the temperature was controlled to ca. 5 °C). The blue line represents the environmental temperature measured at a corner point in the cold-water bath.

Water possesses many interesting properties, for example, its density-temperature dependence between 0 and 4 °C, and its density being higher than that of ice. These result in water freezing at its surface and ice floating on top of the water. As reported in previous work^10^, a surface plasmon resonant band of AuNPs in solution appears at 519 nm. This plasmon band’s center is red-shifted to 538 nm and becomes broader over the entire visible light region when AuNPs are adsorbed onto ceramic particles. This characteristic of localized surface plasmon resonance of AuNPs adsorbed onto ceramic particles indicates the effect of hot electron transfer (HET) for breaking HBs of bulk water, which can be achieved under illumination with white light and further enhanced by wavelength-optimized resonant light. Moreover, the creation and persistence of PAW with reduced HBs were comprehensively discussed in a previous report^15^. In this study, the PAW-based idea of water energy and HBs was applied to prolong the presence of ice layers under normal conditions. Fig. S1 (Supplementary Information, SI) shows schematic descriptions of experiments of freezing and melting water, and for preparing PAW and DI water. Figure 1a demonstrates the temperature-time dependences of PAW and DI water for freezing processes performed in an ice bath of a salt-ice-water system at ca. −20 °C in the beginning. This plot shows the average temperature with time based on three individual replicates of PAW and DI water. Obviously, the freezing time of PAW (14 min) was faster than that of DI water (20 min) under the same cooling conditions. Figure S2a shows three corresponding individual plots (Fig. 1a is the result of average temperature) regarding the temperature-time dependencies of PAW and DI water during the freezing process. In these individual dependencies, the average freezing time for PAW was 12.3 (11, 14, and 12) min and for DI water was 14.7 (13, 20, and 11) min.[must be really high standard deviations?] Compared to DI water, the freezing time of PAW decreased by ca. 16%. The freezing time was calculated by when the stirring bar in the sample tube stopped rotating due to the water having frozen. At the same time, the temperature was maintained at ca. 0 °C. Initially, water does not freeze below 0 °C, meaning that supercooled liquid water is not in thermodynamic equilibrium^25^. Thus, in the freezing process, the temperature of water first decreases to a specific level below 0 °C. Then the temperature gradually increases to ca. 0 °C for freezing. This experimental result suggests that the entire freezing process of the PAW system was faster than that of the DI water system. Figure 1b demonstrates the average temperature-time dependencies of ice from frozen PAW and DI water for melting processes performed in a cold-water bath at ca. 5 °C at the beginning. The three corresponding individual plots are shown in Fig. S2b. Interestingly, compared to DI water (14 min), the melting time of PAW (15 min) increased by ca. 7% under the same heating conditions. This result is consistent with the corresponding melting heat, as discussed below. The melting time was calculated when the stirring bar in the sample tube began rotating due to the ice having melted. At the same time the temperature began rising. The corresponding reproducible experiments of Fig. 1 with similar results are shown in Fig. S3. As shown in the literature^26^, the presence of a nucleus, in which water molecules possess a well-defined arrangement, is the first requirement for the freezing process. For further crystallization, other free water molecules close to the nucleus are adsorbed by HBs onto the formed nucleus. Then, growing structures of nuclei and crystals contribute to the final freezing. Thus, sufficient energy is needed for ordering and adsorption during the freezing process. As reported before^10,15^, the created PAW possesses a reduced HB structure and a higher chemical potential. The former makes more free water molecules available in PAW to bind to other water molecules by HBs. The latter makes ordering and adsorption more efficient. Therefore, a more-rapid freezing process was observed with the PAW system. A smaller specific heat of PAW^15^ also contributes to more-rapid freezing. It is well known that ice has a nearly perfect tetrahedral symmetry around each water molecule connected with strong HBs. Compared to ice from DI water, the slower melting of ice from PAW suggests that the HB network in PAW-based ice was stronger than that in the DI water-based ice. Thus the lattice energy of ice in the PAW system was higher, making it more difficult to melt. Because PAW with reduced HBs is in a high-energy state, it can more easily transform into a stronger HB structure in a low-energy state during cooling for freezing. This should be accompanied by a release of more available energy, resulting in ice with a more-perfect tetrahedral symmetry. Thus, more heat is necessary to melt PAW-based ice.

**Figure 2:**
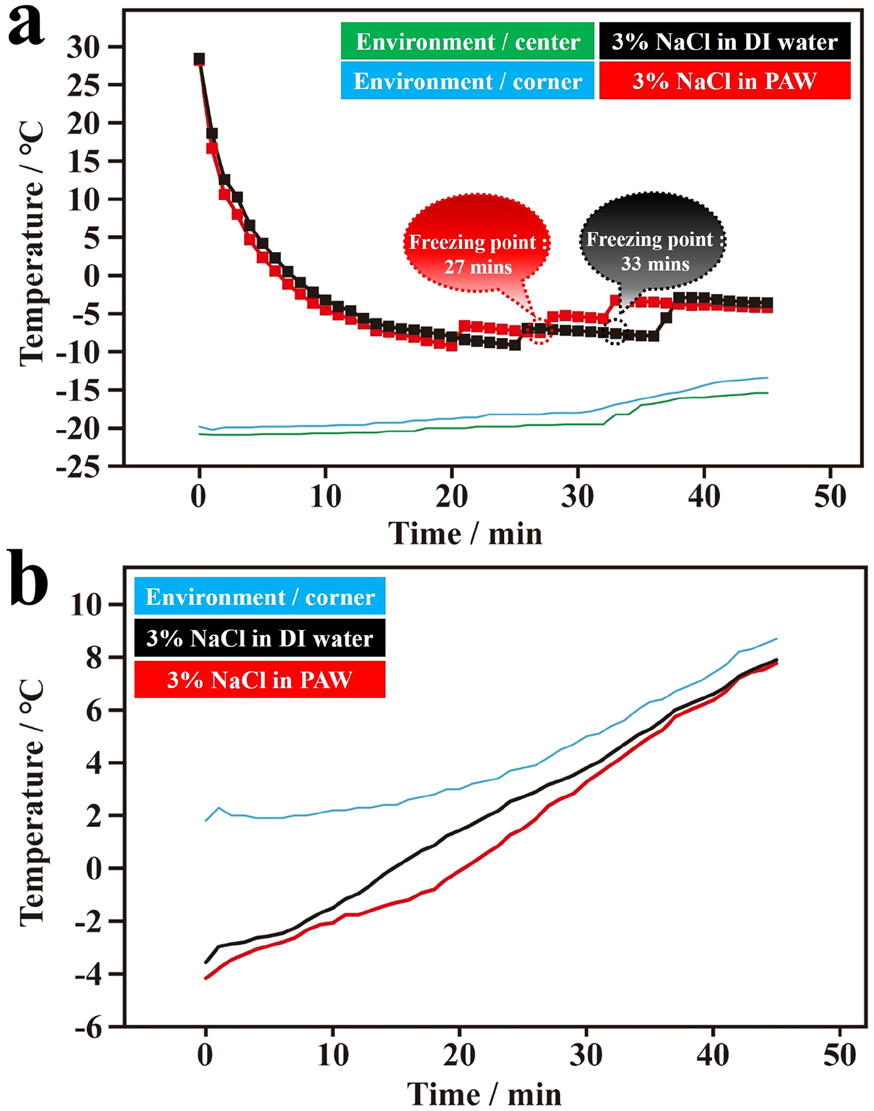
Temperature-time dependencies of plasmon-activated water (PAW) solutions (3 wt% NaCl) and deionized (DI) water solutions (3 wt% NaCl) during freezing and melting processes. (**a**) Temperature-freezing time dependencies of the PAW solution (red line) and DI water solution (black line) in an ice bath of a salt-ice-water system (the temperature was controlled to ca. −20 °C). The green and blue lines represent the environmental temperatures measured at the center point and at a corner point, respectively, in the ice bath. (**b**) Temperature-melting time dependencies of frozen PAW solution (red line) and frozen DI water solution (black line) in a cold water bath (the temperature was controlled to ca. 5 °C). The blue line represents the environmental temperature measured at a corner point in the cold-water bath.

Seawater contains NaCl, KCl, and other electrolytes. To simplify the situation, a 3 wt% NaCl solution was used in this study. Figure 2a shows the average temperature-time dependencies of PAW solutions (3 wt% NaCl) and DI water solutions (3 wt% NaCl) during the freezing process. In the freezing of water solutions to ice, salting out naturally occurs. This salting-out process is accompanied by energy exchange with surrounding water molecules. Correspondingly, this influences the recorded temperature-time dependency of salt water in the freezing process. Thus, several step-like functions were observed due to the phenomenon of salting out. Clearly, the freezing time of PAW solutions was faster than that of the DI water solution under the same cooling conditions, although the freezing process was more complicated compared to that of pure water. Figure S4a shows the three corresponding individual plots (in Fig. 2a) regarding the temperature-time dependencies of PAW solutions and DI water solutions during the freezing process. In these individual dependencies, average freezing times for the PAW solution and DI water solution were 27 (32, 28, and 21) and 34 (26, 38, and 38) min, respectively.[also give standard deviations?] Compared to the DI water solution, the freezing time of the PAW solution decreased by ca. 21%. Figure 2b demonstrates the average temperature-time dependences of ice from the frozen PAW and DI water solutions during the melting process. The three corresponding individual plots are shown in Fig. S4b. Basically, compared to the DI water solution, the melting time of the PAW solution increased, which was qualitatively evaluated from a lower measured temperature at a constant heating time under the same heating conditions.

**Figure 3:**
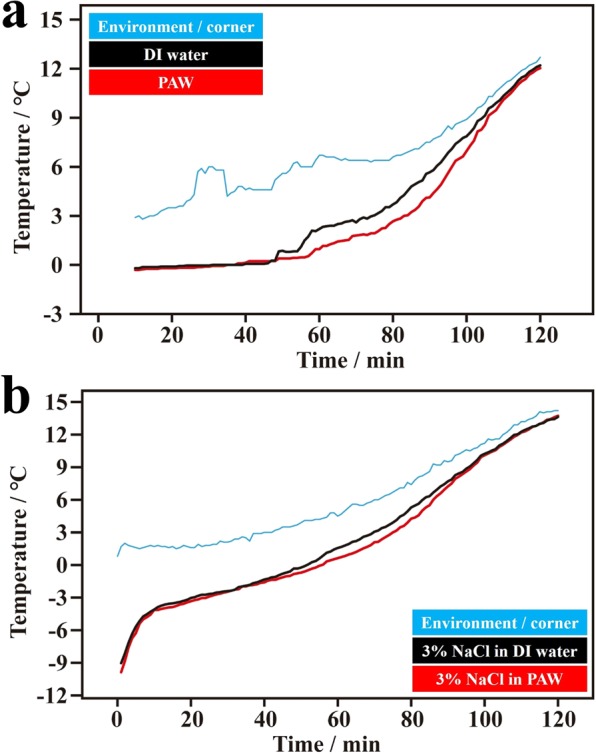
Temperature-melting time dependencies of ice based on completely frozen plasmon-activated water (PAW) and on completely frozen deionized (DI) water, and salty ice based on their completely frozen solutions (3 wt% NaCl) in a cold-water bath (the temperature was controlled to ca. 5 °C) during the melting process. The ice was obtained by placing pure water and salty solutions in a refrigerator at −20 °C. (**a**) Temperature-melting time dependencies of frozen PAW (red line) and frozen DI water (black line). The blue line represents the environmental temperature measured at a corner point in the cold-water bath. (**b**) Temperature-melting time dependencies of the frozen PAW solution (red line) and frozen DI water solution (black line). The blue line represents the environmental temperature measured at a corner point in the cold-water bath.

In further diversified studies, we directly investigated the melting kinetics of completely frozen ice, which was obtained by placing pure water of PAW and DI water, and their salty solutions in a refrigerator at −20 °C overnight. Figure 3 shows the corresponding results. The average temperature was obtained from results of three individual experiments. Basically, compared to the DI water system, melting was slower for ice of both frozen PAW and its solution at a constant heating rate. These interesting results indicate that using the PAW system can prolong the existence of ice layers.

As described in the SI, the prepared PAW contained ca. 0.45 ppb Au and other slightly dissolved metal ions (ca. 4.3 × 10^−6^ N). According to colligative properties, these dissolved impurities would result in freezing-point depression, compared to pure DI water. This means that freezing is harder for water that contains impurities. Therefore, the observed more-rapid freezing of PAW was independent of the slightly dissolved impurities. Moreover, the contribution of impurities in PAW to PAW’s distinct properties was previously excluded^10,15^. We further investigated if directly adding different concentrations of AuNPs (5, 0.5, and 0.05 ppm) to DI water and resonantly illuminating the solution could produce the above-discussed effects as for PAW. The results were negative, as shown in Fig. S5. There was no influence on the speed of freezing or the corresponding melting process for DI water that contained AuNPs.

Compared to DI water, the prepared PAW possesses a reduced HB structure. This distinct property is responsible for a higher evaporation rate in ambient laboratory air, as reported before^10,14^. Figure S7 shows quantities that evaporated with time of fresh PAW and DI water, and the melted ice from frozen PAW and DI water. Basically, the evaporation rates of fresh DI water and melted ice from its frozen DI water were similar. For PAW (from room temperature (RT) to −20 °C, and back to RT again), ca. a 50% higher evaporation rate was maintained, compared to fresh PAW. This result suggests that the prepared PAW is reusable.

### Practical strategy for prolonging the presence of solid ice based on PAW

**Figure 4:**
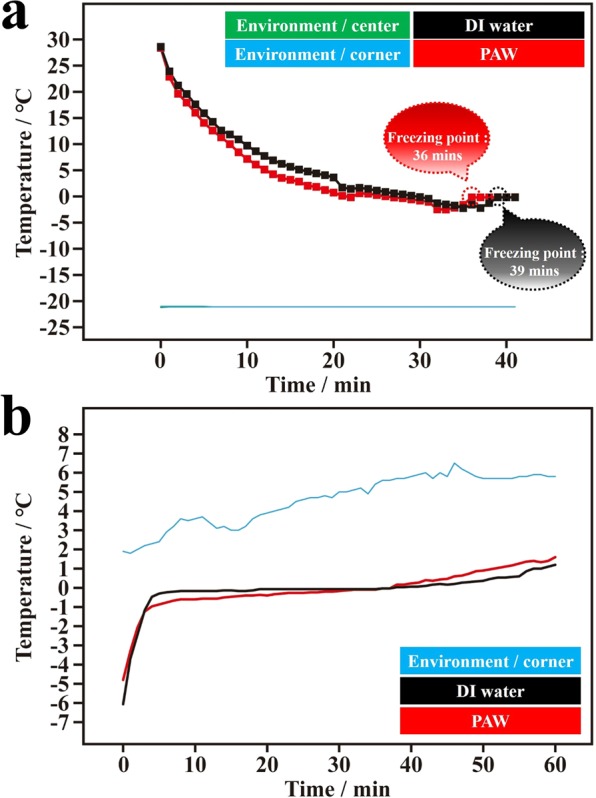
Temperature-time dependencies of plasmon-activated water (PAW) and deionized (DI) water (based on gold nanoparticle (AuNP)-coated filter paper and AuNP-free filter paper, respectively, under illumination by green LEDs in preparation) during freezing and melting processes. (**a**) Temperature-freezing time dependencies of PAW (red line) and DI water (black line) in an ice bath of a salt-ice-water system (the temperature was controlled to ca. −20 °C). The green and blue lines represent the environmental temperatures measured at the center point and at a corner point, respectively, in the ice bath. (**b**) Temperature-melting time dependencies of frozen PAW (red line) and frozen DI water (black line) in a cold-water bath (the temperature was controlled to ca. 5 °C). The blue line represents the environmental temperature measured at a corner point in the cold-water bath.

As discussed above, the PAW system has the potential to prolong the existence of sea ice. However, it is impossible for all seawater to become PAW-based water. Based on the fact that sea ice always floats on the sea and that PAW is created on AuNPs with resonant illumination, we propose a practical strategy of AuNP-coated materials that float on the sea surface. Sunshine contains the necessary resonant green light for exciting AuNPs to produce HET^27,28^ to adjacent seawater, so PAW can be prepared. In the laboratory, we first followed this idea using resonantly illuminated AuNP-coated filter paper to examine the corresponding effects (See SI). Figure 4a shows the corresponding average (of three individual samples) temperature-time dependencies of prepared PAW and DI water during the freezing process. Encouragingly, the results were similar to those discussed in Fig. 1a for typically prepared PAW. Compared to DI water (39 min), the freezing time of PAW (36 min) decreased by ca. 7.7%. A consistent tendency of the temperature-time dependencies (Fig. 1b) was also observed as shown in Fig. 4b for PAW prepared using AuNP-coated filter paper. During the melting process, the PAW system still melted more slowly, although the temperature rose more quickly after the temperature exceeded 0 °C. Figure S6 shows the reproducible experiments of Fig. 4b. The results were similar.

**Figure 5:**
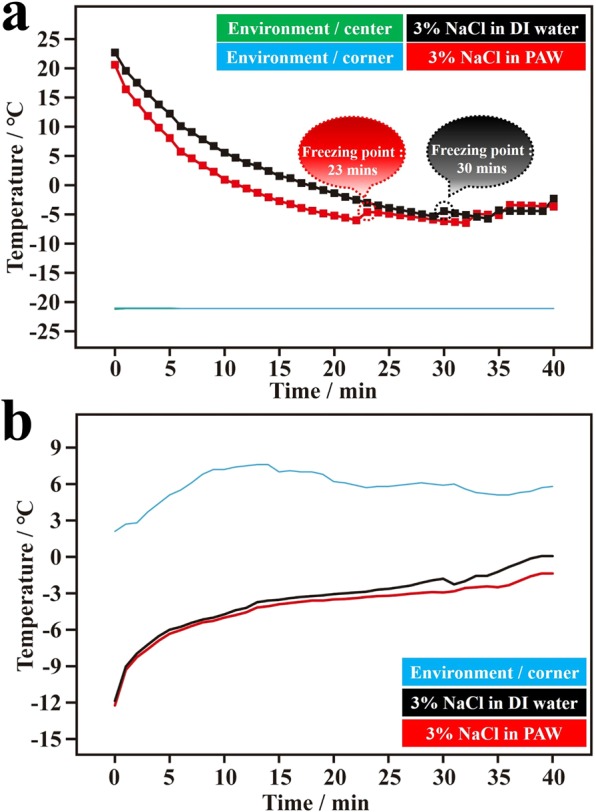
Temperature-time dependencies of plasmon-activated water (PAW) solutions (3 wt% NaCl) and deionized (DI) water solutions (3 wt% NaCl) (based on gold nanoparticle (AuNP)-coated filter paper and AuNP-free filter paper, respectively, under illumination with green LEDs in preparations) during freezing and melting processes. (**a**) Temperature-freezing time dependencies of the PAW solution (red line) and DI water solution (black line) in an ice bath of a salt-ice-water system (the temperature was controlled to ca. −20 °C). The green and blue lines represent environmental temperatures measured at the center point and at a corner point, respectively, in the ice bath. (**b**) Temperature-melting time dependencies of the frozen PAW solution (red line) and frozen DI water solution (black line) in a cold-water bath (the temperature was controlled at ca. 5 °C). The blue line represents the environmental temperature measured at a corner point in the cold-water bath.

Figure 4 shows the results based on pure water. Similar experiments were performed using 3 wt% NaCl solutions, as exhibited in Fig. 5. Compared to DI water solutions (30 min), the average freezing time of PAW solutions (23 min) was ca. 23% shorter, as shown in Fig. 5a. During the melting process, the PAW solution system melted more slowly as the temperature increase was delayed, as shown in Fig. 5b.

### Analyses of melting heats of PAW and DI water

**Figure 6:**
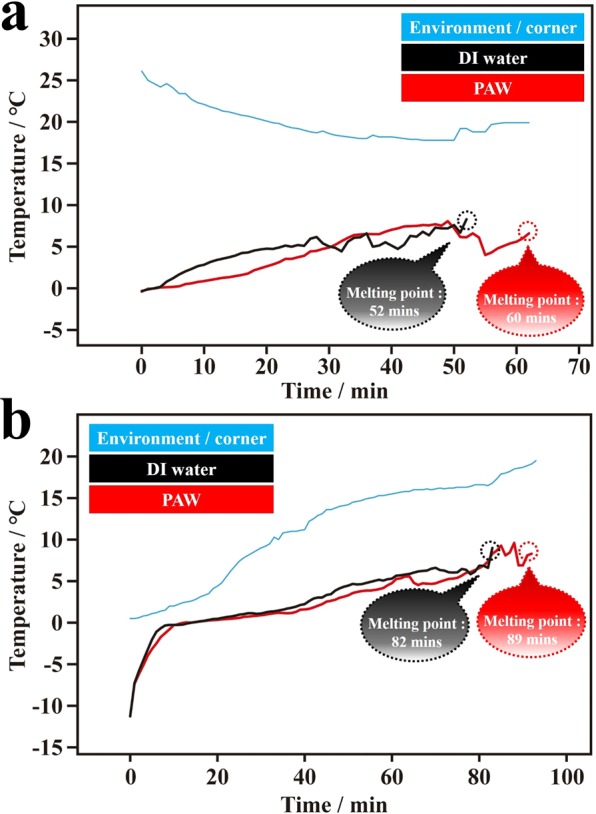
Temperature-melting time dependencies of ice based on completely frozen plasmon-activated water (PAW) and on completely frozen deionized (DI) water in a stainless steel plate with cold water (at ca. 25 and 0 °C at the beginning) on a heater (see Supplementary Information for details). The ice was obtained by placing pure water in a refrigerator at −20 °C. (**a**) Temperature-melting time dependencies of frozen PAW (red line) and frozen DI water (black line). The blue line represents the environmental temperature measured in the cold water in the stainless steel plate. The temperature of the cold water was ca. 25 °C at the beginning. (**b**) Temperature-melting time dependencies of frozen PAW (red line) and frozen DI water (black line). The blue line represents the environmental temperature measured in the cold water in the stainless steel plate. The temperature of the cold water was ca. 0 °C at the beginning.

**Figure 7:**
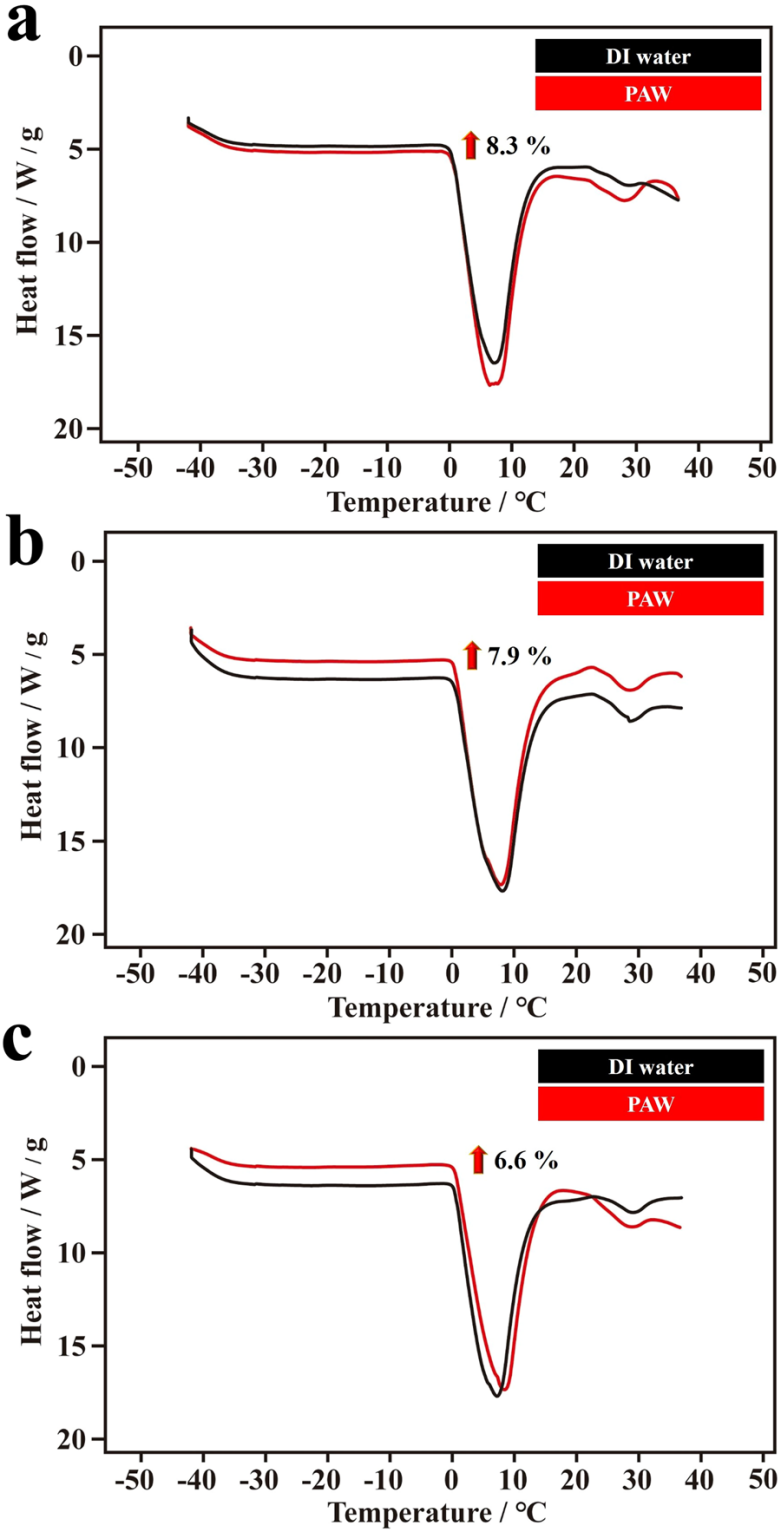
DSC thermodynamic responses of plasmon-activated water (PAW) and deionized (DI) water. The experiments were performed under the same heating rate of 10 °C min^−1^ from −40 to 40 °C.

Compared to the DI water system, frozen PAW melted with greater difficulty, which was reflected in the higher melting heat due to stronger HBs in the frozen PAW. The HBs in PAW were reduced; however, HBs in frozen PAW were stronger. This phenomenon is very interesting. In this work, the melting heat was qualitatively evaluated by two methods based on the well-known melting heat of DI water. One method was designed in our laboratory. The other one was based on instrumental differential scanning calorimetry (DSC). Figure 6a shows the average temperature-melting time dependencies of ice based on completely frozen PAW and completely frozen DI water in a stainless steel plate with cold water (at ca. 25 °C at the beginning) on a heater. The recording of temperature-time stopped when the ice had completely melted, which was decided by naked-eye observation. Compared to frozen DI water (average 52 min; 50, 50, and 56 min), the average melting time of frozen PAW (average 60 min; 55, 61, and 63 min) was ca. 15% longer. Figure 6b shows similar results for the experiment performed in a stainless steel plate with colder water (at ca. 0 °C at the beginning) on a heater. Compared to frozen DI water (average 82 min; 84, 50, and 85 min), the average melting time of frozen PAW (average 89 min; 88, 86 and 93 min) was ca. 8.5% longer. The results suggest that the melting heat of frozen PAW is higher than that of the frozen DI water, because frozen PAW possesses stronger HBs. Moreover, we employed instrumental analyses to further confirm these interesting properties. The characteristic DSC heating thermograms of PAW and DI water are exhibited in Fig. 7 for three individual samples. The melting heat was obtained by integrating the peak area (from 0 to 15 °C for every sample) in the DSC thermogram. Then, the integrated area was divided by the mass of water used. As shown in Fig. 7, compared to the DI water systems, the melting heats of the PAW systems were 8.3%, 7.9%, and 6.6% higher, for an average 7.6% increase. Figure S8 shows reproducible experiments based on five individual samples. Similarly, compared to DI water systems, the average melting heat of the PAW system increased by 13%. If the largest and the smallest values were deleted from the five individual samples, the average melting heat of the PAW system increased by 11%.

As discussed in our previous reports^10,15^, the prepared stable PAW exhibits distinct properties at room temperature, which significantly differ from the properties of untreated bulk water, examples being their significantly negatively charged properties and higher solubilities of solutes. To further confirm the plasmonic effect on the preparation of PAW with distinct properties compared to DI water, zeta potentials of PAW and DI water, and saturated solubilities of NaCl in them under ambient laboratory air were measured. As shown in Fig. S9a for group I, the zeta potential of PAW was −30.3 ± 0.65 mV, while DI water was close to electronically neutral (−0.18 ± 0.21 mV). Similarly, for group II, the zeta potential of PAW was −29.1 ± 0.57 mV, while DI water was also close to electronically neutral (−1.25 ± 0.23 mV). Water samples in groups I and II corresponded to water samples discussed in Fig. 1. Moreover, as shown in Fig. S9b for group I, the saturated solubility of NaCl in DI water at room temperature was 35.8 ± 1.2 g dL^−1^, which is very close to the well-known value of 36.0 g dL^−1^ at 25 °C. Interestingly, this saturated solubility was 43.9 ± 2.3 g dL^−1^ in PAW. It is an increase of 23% compared to the value measured in DI water. Similarly, for group II, the saturated solubility of NaCl in DI water at room temperature was 36.1 ± 1.3 g dL^−1^. The saturated solubility was 43.5 ± 1.9 g dL^−1^ in PAW, for an increase of 20% compared to the value measured in DI water.

In summary, we innovatively utilized the modified PAW to prolong existing ice layers, compared to DI water, via a two-pronged approach of more-rapid freezing and slower melting. Also, a practical strategy to prolong the presence of solid ice based on PAW was proposed. Because the hydrogen-bonded network of water is dynamic, we performed various reproducible experiments to confirm the success of these proposed methods. These efforts would make it possible to save polar bears by maintaining their sea-ice habitat in spite of increasing global warming. We believe these new concepts could lead to a variety of applications in multiple fields.

## Methods

### Chemicals and materials

NaCl was purchased from Sigma-Aldrich Organics. All of the pure water and solutions were prepared using deionized (DI) 18.2 MΩ cm water provided by a Milli-Q system. All of the experiments were performed in an air-conditioned room at ca. 24 °C. The water temperature was ca. 24.1 °C.

### Preparation of PAW

The preparation conditions were previously reported^10^. In a typical preparation, DI water (pH 7.15, temperature of 24.1 °C) flowed through a glass tube filled with AuNPs adsorbed onto ceramic particles under illumination with green light-emitting diodes (LEDs, with a wavelength maximum centered at 530 nm). Then the PAW (pH 7.15, temperature of 24.1 °C) was collected in glass sample bottles for subsequent use as soon as possible. Detailed methods are shown in Supplementary Information (SI).

## Supplementary information

Plasmon-Activated Water Can Prolong Existing Sea-Ice Habitats to Potentially Save Polar Bears

## References

[1] Shultz MJ, Vu TH, Meyer B, Bisson P (2012). Water: a responsive small molecule. Accounts Chem. Res..

[2] Fayer MD (2012). Dynamics of water interacting with interfaces, molecules, and ions. Accounts Chem. Res..

[3] Fenn EE, Wong DB, Fayer MD (2009). Water dynamics at neutral and ionic interfaces. Proc. Natl. Acad. Sci..

[4] Kumar M, Li H, Zhang X, Zeng XC, Francisco JS (2018). Nitric acid-amine chemistry in the gas phase and at the air-water interface. J. Am. Chem. Soc..

[5] Liu J, He X, Zhang JZH, Qi LW (2018). Hydrogen-bond structure dynamics in bulk water: insights from: ab initio simulations with coupled cluster theory. Chem. Sci..

[6] Chaban VV, Prezhdo VV, Prezhdo OV (2012). Confinement by carbon nanotubes drastically alters the boiling and critical behavior of water droplets. ACS Nano.

[7] Tunuguntla RH (2017). Enhanced water permeability and tunable ion selectivity in subnanometer carbon nanotube porins. Science.

[8] Davis JG, Rankin BM, Gierszal KP, Ben-Amotz D (2013). On the cooperative formation of non-hydrogen-bonded water at molecular hydrophobic interfaces. Nat. Chem..

[9] Jiménez-Ángeles F, Firoozabadi A (2018). Hydrophobic hydration and the effect of nacl salt in the adsorption of hydrocarbons and surfactants on clathrate hydrates. ACS Cent. Sci..

[10] Chen HC (2014). Active and stable liquid water innovatively prepared using resonantly illuminated gold nanoparticles. ACS Nano.

[11] Chen HC (2014). Innovative strategy with potential to increase hemodialysis efficiency and safety. Sci. Rep..

[12] Chen HC (2015). Quantitative evaluation on activated property-tunable bulk liquid water with reduced hydrogen bonds using deconvoluted raman spectroscopy. Anal. Chem..

[13] Hwang BJ (2015). Innovative strategy on hydrogen evolution reaction utilizing activated liquid water. Sci. Rep..

[14] Yang CP (2015). Effective energy transfer via plasmon-activated high-energy water promotes its fundamental activities of solubility, ionic conductivity, and extraction at room temperature. Sci. Rep..

[15] Chen HC (2016). Creation of electron-doping liquid water with reduced hydrogen bonds. Sci. Rep..

[16] Chen HC (2016). Environmentally friendly etching agent: vapor from hot electron-activated liquid water. Green Chem..

[17] Chen HC (2016). Triggering comprehensive enhancement in oxygen evolution reaction by using newly created solvent. Sci. Rep..

[18] Chen HC (2016). Multifunctions of excited gold nanoparticles decorated artificial kidney with efficient hemodialysis and therapeutic potential. ACS Appl. Mater. Interfaces.

[19] Chen HC (2018). *In situ* and real-time reduction of water molecules’ interaction for efficient water evaporation. Desalination.

[20] Chen HC (2018). Plasmon-activated water effectively relieves hepatic oxidative damage resulted from chronic sleep deprivation. RSC Adv..

[21] Wang CK (2018). Innovatively therapeutic strategy on lung cancer by daily drinking aantioxidative plasmon-induced activated water. Sci. Rep..

[22] Jin J, Goddard WA (2015). Mechanisms underlying the mpemba effect in water from molecular dynamics simulations. J. Phys. Chem. C.

[23] Pyrgiotakis G (2015). Inactivation of foodborne microorganisms using engineered water nanostructures (EWNS). Environ. Sci. Technol..

[24] Pyrgiotakis G (2016). Optimization of a nanotechnology based antimicrobial platform for food safety applications using engineered water nanostructures (EWNS). Sci. Rep..

[25] Gottke SD, Brace DD, Hinze G, Fayer MD (2001). Time domain optical studies of dynamics in supercooled *o*-terphenyl: Comparison to mode coupling theory on fast and slow time scales. J. Phys. Chem. B.

[26] Esposito S, De Risi R, Somma L (2007). Mpemba effect and phase transitions in the adiabatic cooling of water before freezing. Physica A.

[27] Simoncelli S, Li Y, Cortés E, Maier SA (2018). Imaging plasmon hybridization of fano resonances via hot-electron-mediated absorption mapping. Nano Lett..

[28] Xu J (2018). CdS core-Au plasmonic satellites nanostructure enhanced photocatalytic hydrogen evolution reaction. Nano Energy.

