# Supplementary material for: Plasmon-Activated Water can Prolong Existing Sea-Ice Habitats to Potentially Save Polar Bears
Source: Sci Rep. 2019 Jul 18;9:10398. doi: 10.1038/s41598-019-46867-5 (PMC6639346; doi:10.1038/s41598-019-46867-5)
Supplement: Supplementary file 1 — Plasmon-Activated Water Can Prolong Existing Sea-Ice Habitats to Potentially Save Polar Bears [file 41598_2019_46867_MOESM1_ESM.docx]

**Supplementary Information:**

**Plasmon-Activated Water Can Prolong Existing Sea-Ice Habitats to Potentially Save Polar Bears**

Chih-Ping Yang,^1^ Yi-Hao Wu,^1^ Hui-Yen Tsai,^1^ Jen-Chang Yang,^2^ Yu-Chuan Liu^1,3^

^1^Department of Biochemistry and Molecular Cell Biology, School of Medicine, College of Medicine, Taipei Medical University, No. 250, Wuxing St., Taipei 11031, Taiwan

^2^Graduate Institute of Nanomedicine and Medical Engineering, College of Biomedical Engineering, Taipei Medical University, No. 250, Wuxing St., Taipei 11031, Taiwan

^3^Cell Physiology and Molecular Image Research Center, Wan Fang Hospital, Taipei Medical University

Correspondence and requests for materials should be addressed to Y.-C.L. (email: liuyc@tmu.edu.tw)

**Materials and Methods**

**Preparation of plasmon-activated water (PAW).** The preparation conditions were previously reported^1^. In a typical preparation, deionized (DI) water (pH 7.15, temperature of 24.1 °C) flowed through a glass tube filled with gold nanoparticles (AuNPs) adsorbed onto ceramic particles under illumination with green light-emitting diodes (LEDs, with a wavelength maximum centered at 530 nm). Then the PAW (pH 7.15, temperature of 24.1 °C) was collected in glass sample bottles for subsequent use as soon as possible. Also, another method was used to prepare PAW based on AuNP-coated filter paper. Before preparation, AuNP-coated filter paper (six semicircular pieces of paper; model 5C/55 mm, Advantec) was adsorbed onto the inner wall of a plastic tube (50 mL) with 30 mL of DI water. The adsorbed paper was positioned so that it overlapped as little as possible. During preparation, the sealed experimental tubes were placed on a platform of an orbital shaker, operating at 150 rpm, under illumination with green LEDs for 4 h. Similar experiments were performed, but using AuNP-free filter paper for reference. To further examine the purity of the prepared PAW, inductively coupled plasma-mass spectrometric (ICP-MS) analyses indicated that the concentrations of the slightly dissolved metals in the PAW based on a previous method^1^ were ca. 0.45 ppb for Au, 63 ppb for Na, 35 ppb for K, 18 ppb for Al, 20 ppb for Mg, 2.5 ppb for Ca, and 0.92 ppb for Fe. Excluding Au, the total equivalent molar concentration of these dissolved metals was equal to ca. 4.3 × 10^-6^ N. This measured value was ca. 3.1 × 10^-7^ N for DI water as a reference. Also, the slightly dissolved Au and the total equivalent molar concentration of other dissolved metals in the PAW (based on AuNP-coated filter paper) were 0.68 ppb and 6.5 × 10^-7^ N, respectively. This measured value for the total equivalent molar concentration of other dissolved metals in DI water (based on AuNP-free filter paper) was ca. 4.7 × 10^-7^ N as a reference.

**Freezing process of water (or solutions) in ambient laboratory air.** As shown in Figure S1a, three individual samples of PAW, DI water, and their NaCl-containing solutions (30 mL water, a stirring bar, and a thermometer in a 50 mL-plastic tube) were placed in an ice bath of a salt-ice-water system (297 g NaCl in 1000 g ice, with the temperature set to ca. -20 °C). Corresponding temperatures were recorded every 1 min. In these experiments, the speed of the magnetic stirrer was set to 300 rpm.

**Melting process of ice in ambient laboratory air.** As shown in Figure S1b, three individual samples of ice from PAW, DI water, and their NaCl-containing solutions were placed in a cold water bath (with the temperature set to ca. 5 °C). The corresponding temperatures were recorded every 1 min. Corresponding samples of ice were prepared by moving the entire experimental sets of plastic tubes to a refrigerator at ca. -20 °C overnight after the freezing experiments described above. In these experiments, the speed of the magnetic stirrer was set to 300 rpm.

**Melting process of ice on a heater in ambient laboratory air.** Three individual samples of ice from PAW and DI water were placed in a 250-mL beaker. Then all samples were placed in a stainless steel plate with cold water (at ca. 25 or 0 °C at the beginning) on a heater with a constant heating rate. Corresponding temperatures were recorded every 1 min. Corresponding samples of ice were prepared by placing PAW and DI water (20 mL water, a stir bar, and a thermometer in a 50-mL glass bottle) in a refrigerator at ca. -20 °C overnight. In these experiments, the speed of the magnetic stirrer was set to 300 rpm.

**Melting heat of ice.** Melting heats of ice based on three individual samples from PAW and DI water were measured by differential scanning calorimetry (DSC, Perkin Elmer, Jade DSC) at a heating rate of 10 °C min^-1^ from -40 to 40 °C. Before the DSC measurements, liquid samples (ca. 8 mg) were individually filled into aluminum ingots. The melting heat was obtained by integrating the peak area (from 0 to 15 °C for every sample) in the DSC thermogram and then dividing by the mass of the water used.

**Evaporation rate of melted PAW at room temperature into ambient laboratory air.** Before the experiments, fresh PAW (based on a previous method)^1^ and DI water (30 mL water in 50-mL plastic tubes) were frozen in a refrigerator at -20 °C overnight. Then the frozen water in sealed tubes was placed in a water bath at room temperature to be melted and raised to room temperature to further measure evaporation rates at 1 atm and room temperature. In the experiments, samples of 10 mL of fresh and melted water were added to open glass sample bottles (20 mL), which were placed on a platform of an orbital shaker, operating at 150 rpm. The weight of each glass sample bottle was measured at 0.5, 1, and 2 h to determine the evaporating masses (g) of PAW and DI water. In these experiments, the relative humidity (RH) was ca. 45%. Replicate measurements based on three similar samples were performed. Errors were obtained from the relative standard deviation (RSD).

**Measurement of the zeta potential of water.** Zeta potentials of water samples (600 µL) were analyzed using a Malvern Zetasizer Nano ZS zeta potential analyzer. To avoid the influence of bacteria on the zeta potential during storage, samples were filtered through a membrane with a 0.22-µm pore size to remove bacteria. Filtered water was divided into 3 parts (*n*=3 for each measurement). The parafilm-sealed samples were placed in a dark atmosphere before the measurement.

**Saturated solubility of NaCl in water**. The solubility of NaCl in water was obtained by dissolving excess NaCl in 20 mL of water under stirring for 30 min. Then the solution was placed without stirring for another 30 min. Subsequently, five samples of 1 mL of the clear NaCl-saturated solution were weighed. The saturated solubility of NaCl based on 1 dL water was calculated by utilizing the known densities of 2.165 and 1 g cm^-3^ for NaCl and water, respectively.

**References**

1. Chen, H. C. *et al*. Active and stable liquid water innovatively prepared using resonantly illuminated gold nanoparticles. *ACS Nano* **8**, 2704–2713 (2014).


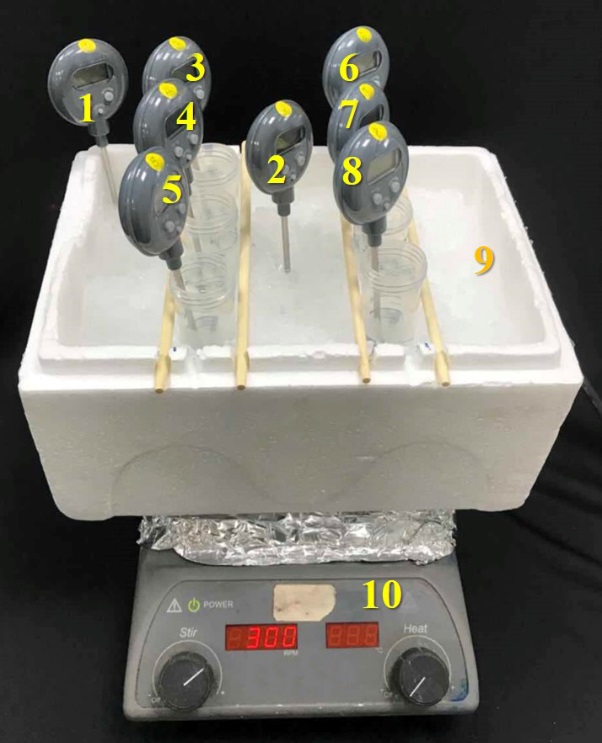


(a)


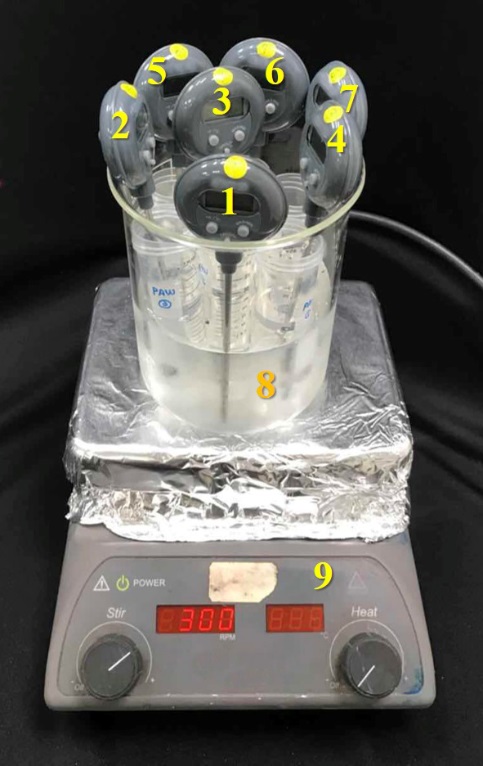


(b)


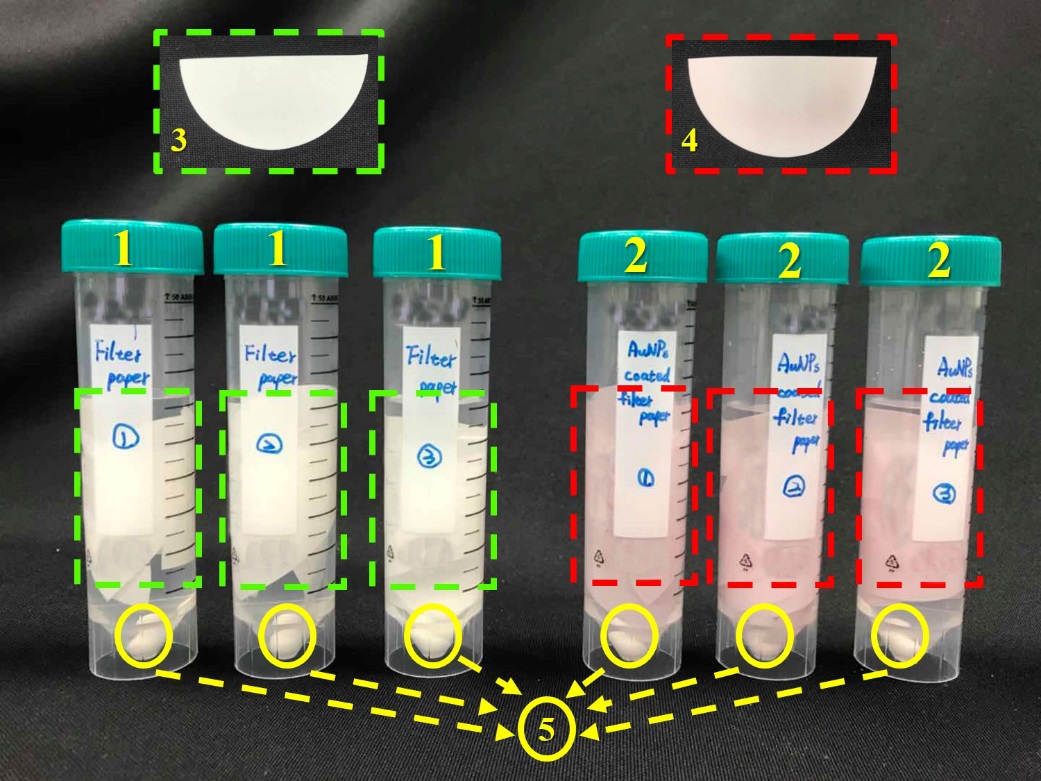


(c)

Figure S1. Schematic descriptions of experiments of freezing and melting water, and for preparations of plasmon-activated water (PAW) and deionized (DI) water (based on gold nanoparticle (AuNP)-coated filter paper and AuNP-free filter paper, respectively, under illumination with green LEDs in preparations). (a) During the freezing process 1 and 2: temperatures measured at a corner point (1) and at the center point (2), respectively, in an ice bath; 3, 4, and 5: temperatures measured in DI water; 6, 7, and 8: temperatures measured in PAW; 9: ice bath of the salt-ice-water system; 10: magnetic stirrer (at 300 rpm). (b) During the melting process 1: temperature measured at the center point in a cold-water bath; 2, 3, and 4: temperatures measured in DI water; 5, 6, and 7: temperatures measured in PAW; 8: cold-water bath; 9: magnetic stirrer (at 300 rpm). (c) 1: DI water prepared using AuNP-free filter paper; 2: PAW prepared using AuNP-coated filter paper; 3: AuNP-free filter paper; 4: AuNP-coated filter paper; 5: stirring bar.


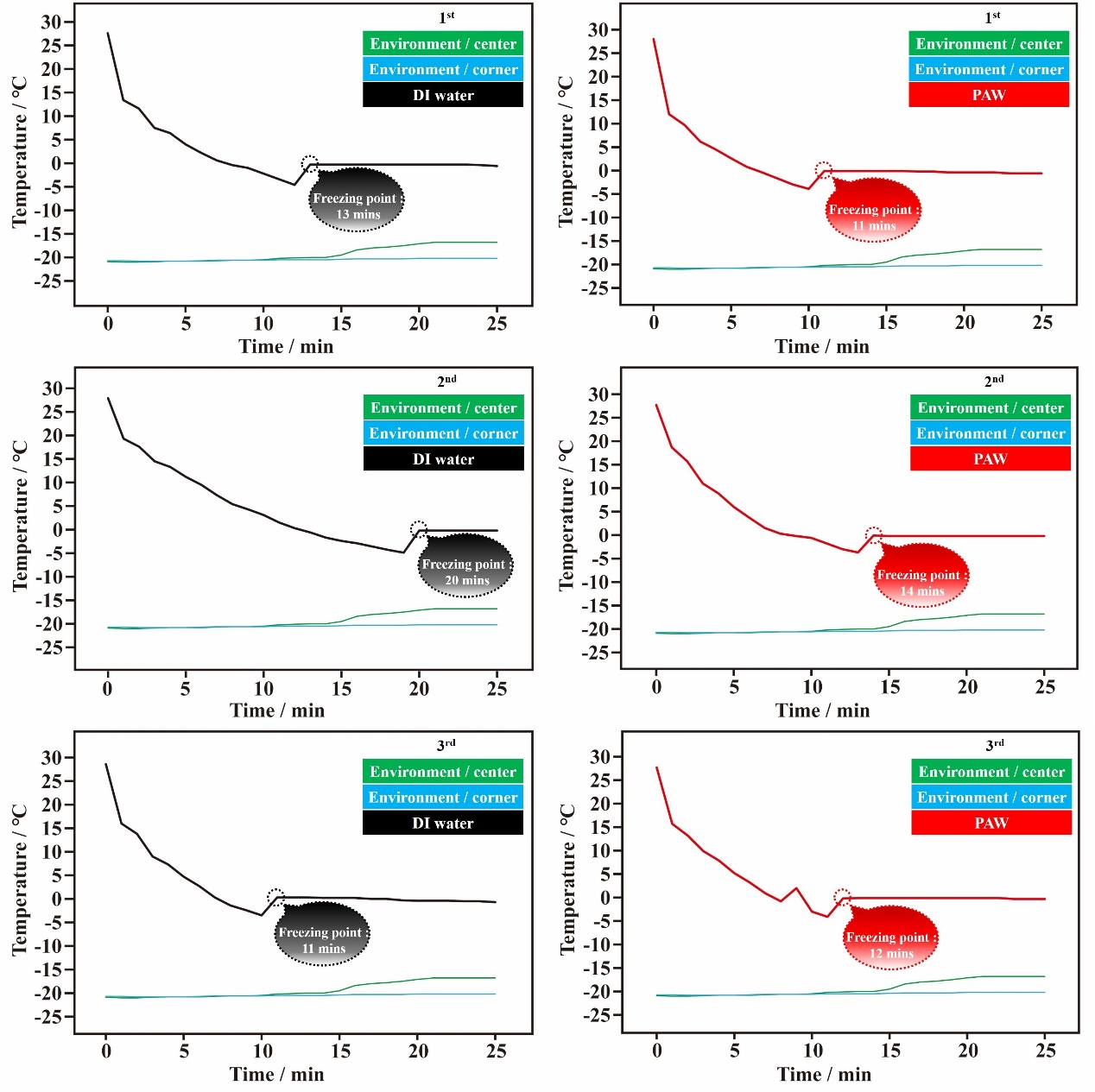


(a)


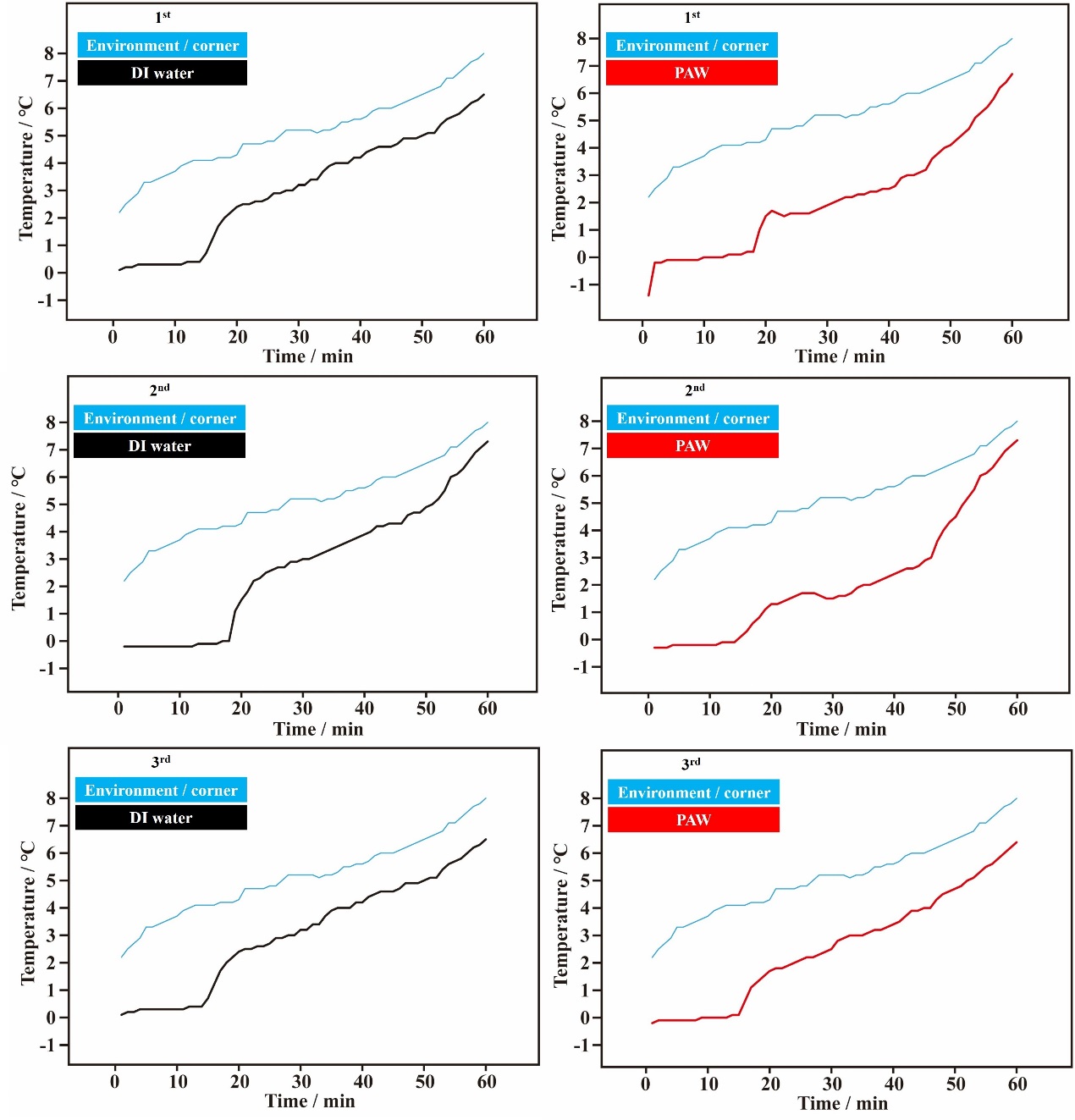


(b)

Figure S2. Individual plots (Figure 1 in the text) regarding temperature-time dependencies of plasmon-activated water (PAW) and deionized (DI) water during freezing and melting processes. (a) Temperature-freezing time dependencies of PAW (red line) and DI water (black line) in an ice bath of a salt-ice-water system (the temperature was controlled to ca. -20 °C). The green and blue lines represent environmental temperatures measured at the center point and at a corner point, respectively, in the ice bath. (b) Temperature-melting time dependencies of frozen PAW (red line) and frozen DI water (black line) in a cold-water bath (the temperature was controlled to ca. 5 °C). The blue line represents the environmental temperature measured at a corner point in the cold-water bath.


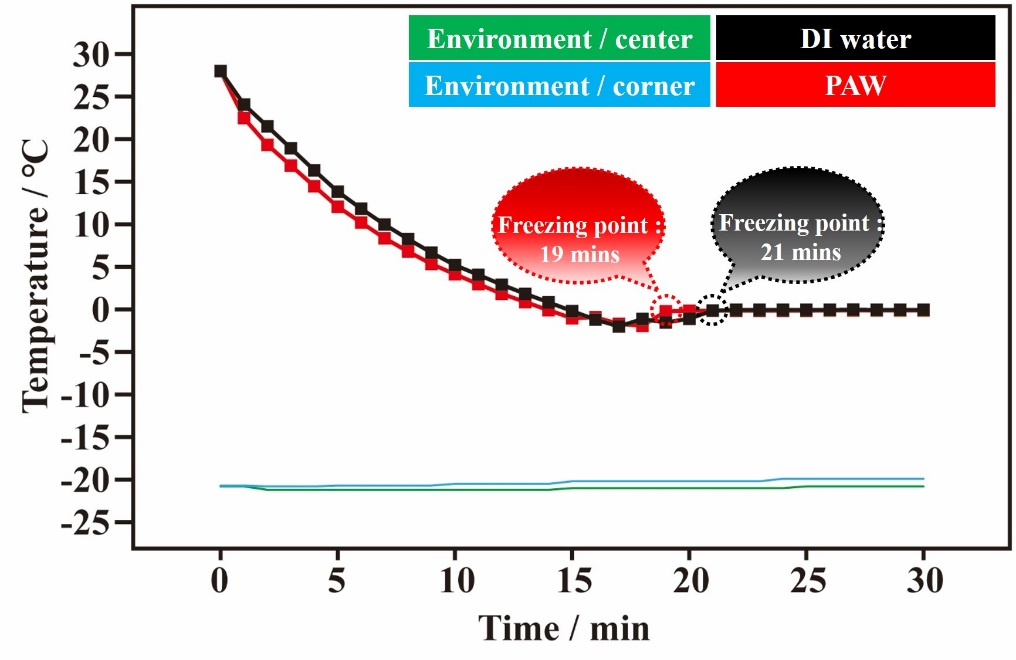


(a)


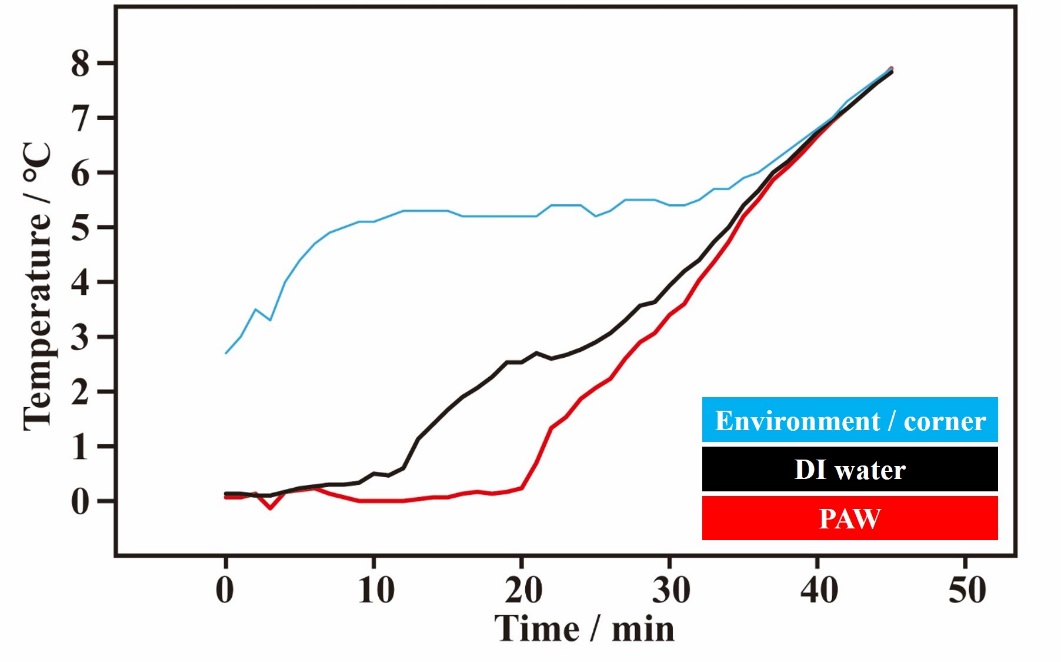


(b)

Figure S3. Reproducible experiments (Figure 1 in the text) regarding temperature-time dependencies of plasmon-activated water (PAW) and deionized (DI) water during freezing and melting processes. (a) Temperature-freezing time dependencies of PAW (red line) and DI water (black line) in an ice bath of a salt-ice-water system (the temperature was controlled to ca. -20 °C). The green and blue lines represent environmental temperatures measured at the center point and at a corner point, respectively, in the ice bath. (b) Temperature-melting time dependencies of frozen PAW (red line) and frozen DI water (black line) in a cold-water bath (the temperature was controlled to ca. 5 °C). The blue line represents the environmental temperature measured at a corner point in the cold-water bath.


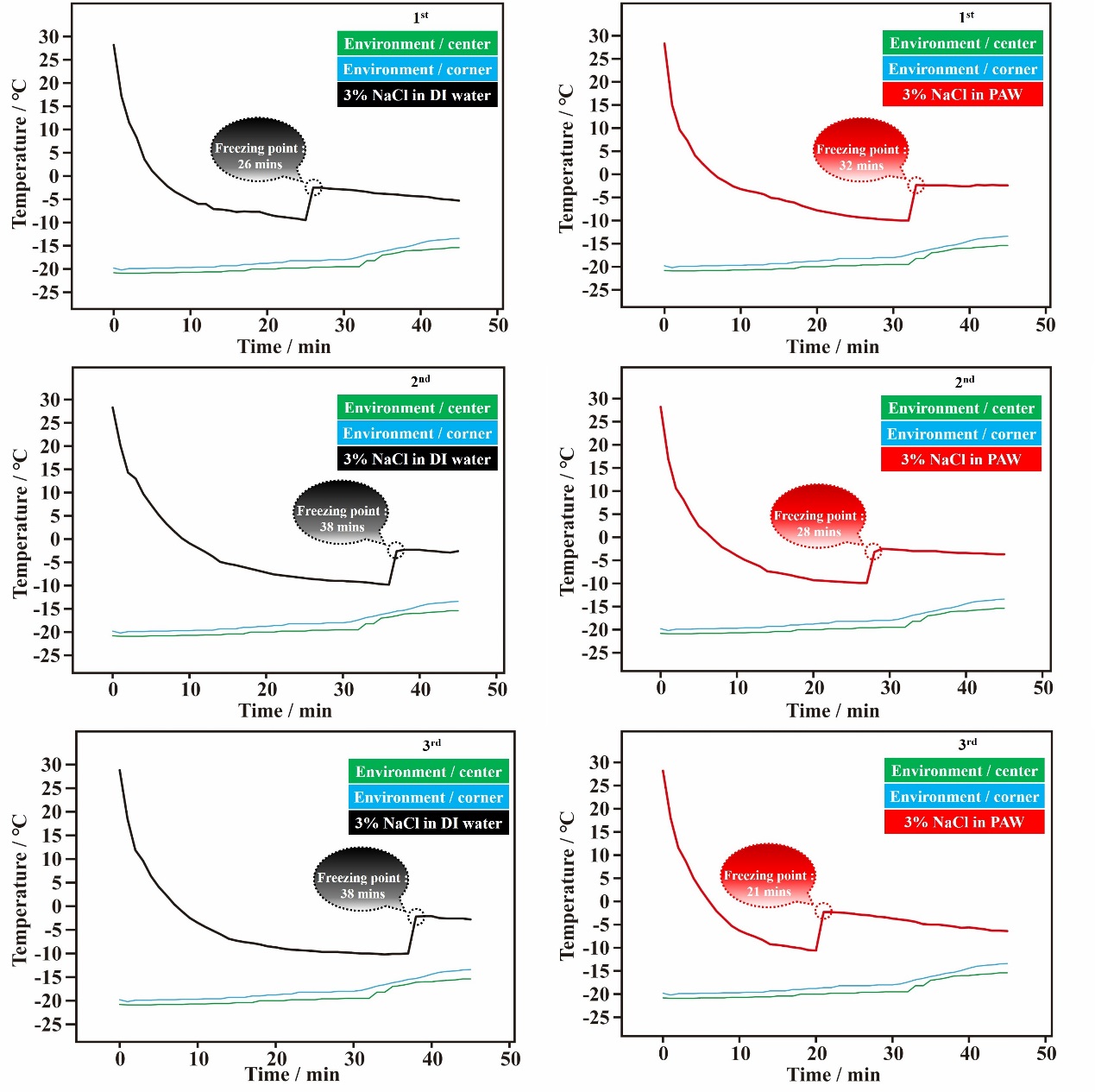


(a)


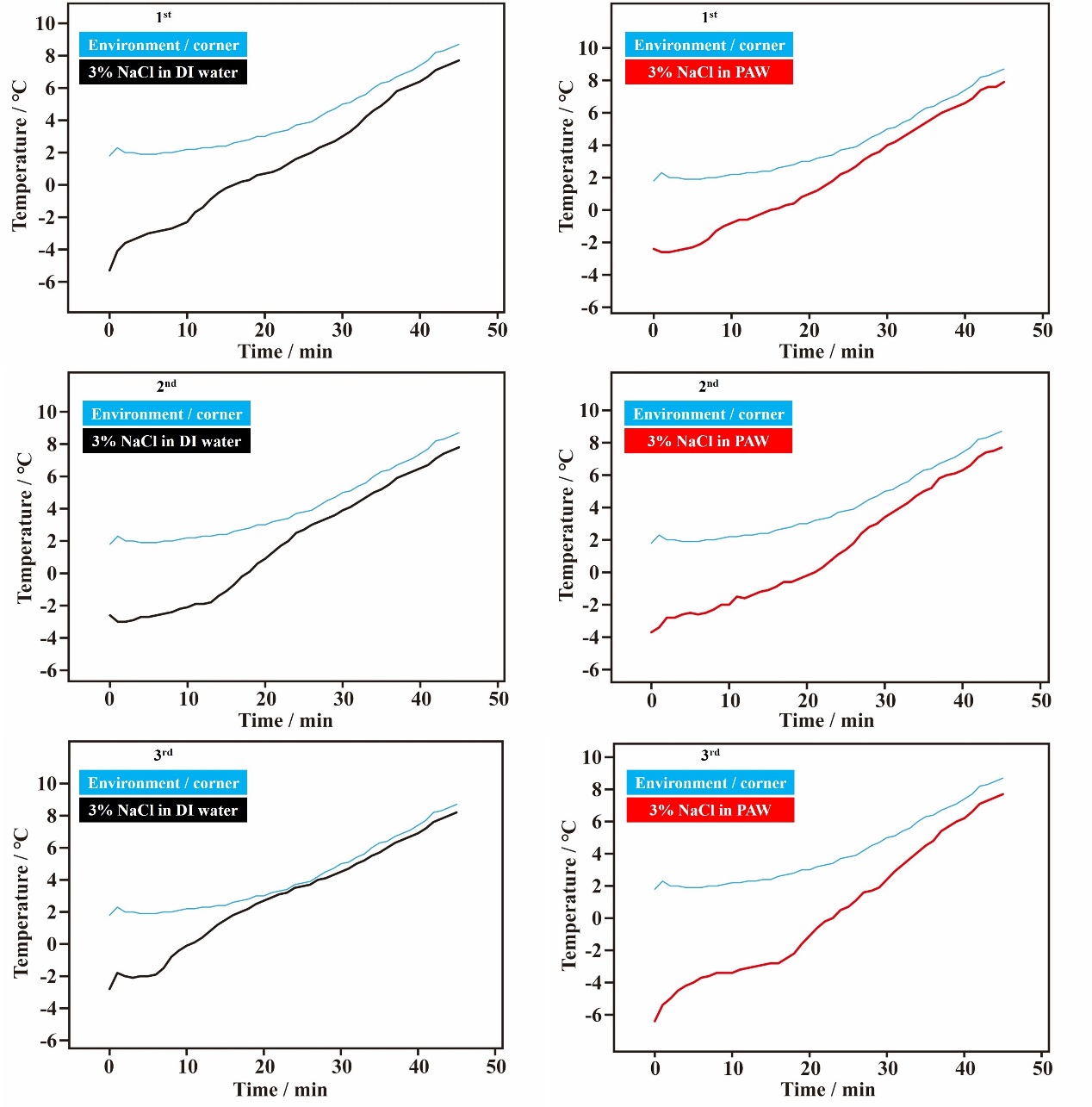


(b)

Figure S4. Individual plots (Figure 2 in the text) regarding temperature-time dependencies of plasmon-activated water (PAW) solutions (3 wt% NaCl) and deionized (DI) water solutions (3 wt% NaCl) during freezing and melting processes. (a) Temperature-freezing time dependencies of the PAW solution (red line) and DI water solution (black line) in an ice bath of a salt-ice-water system (the temperature was controlled to ca. -20 °C). The green and blue lines represent environmental temperatures measured at the center point and at a corner point, respectively, in the ice bath. (b) Temperature-melting time dependencies of the frozen PAW solution (red line) and frozen DI water solution (black line) in a cold-water bath (the temperature was controlled to ca. 5 °C). The blue line represents the environmental temperature measured at a corner point in the cold-water bath.


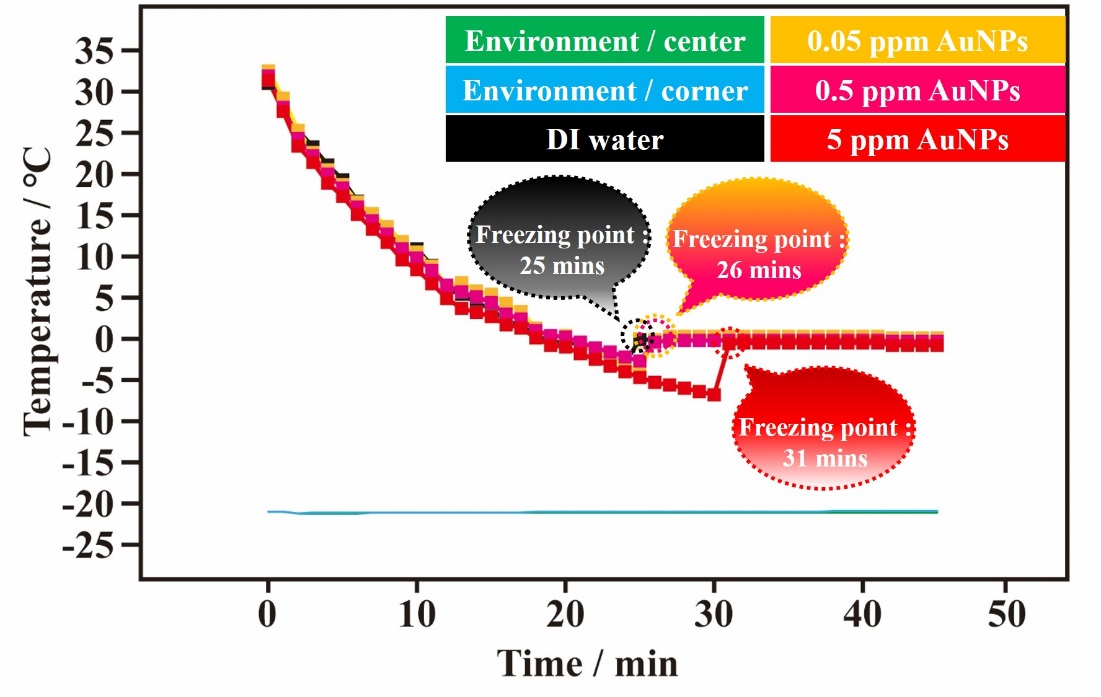


(a)


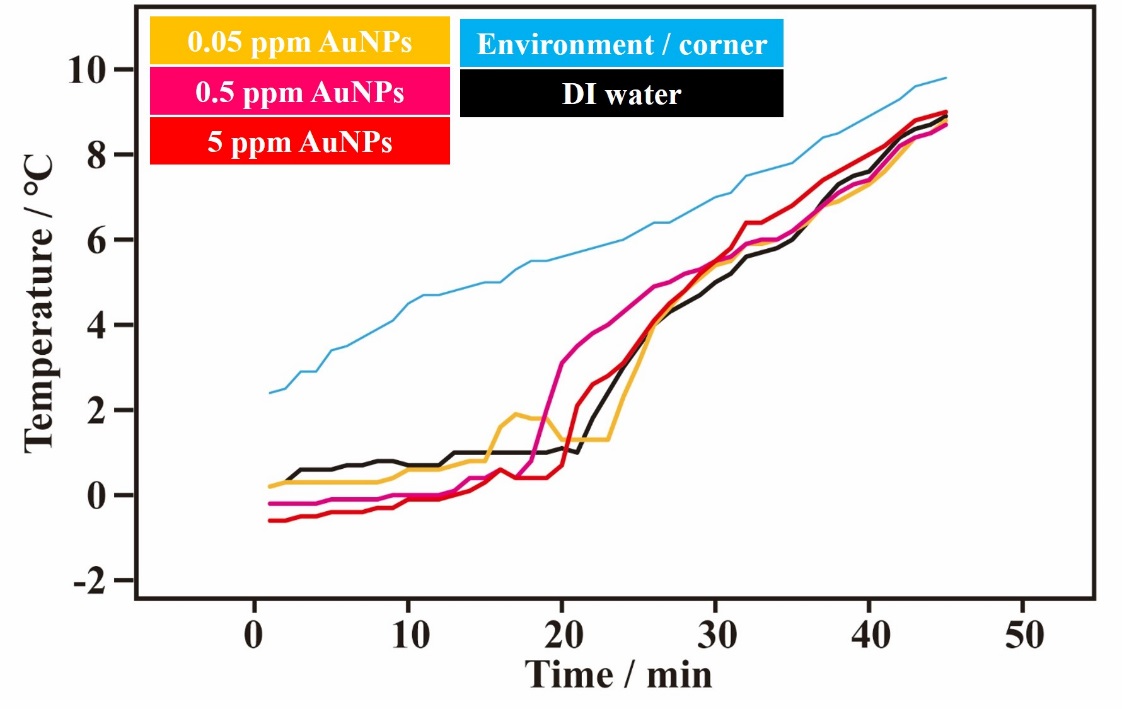


(b)

Figure S5. Temperature-time dependencies of deionized (DI) water and gold nanoparticle (AuNP)-containing DI water during freezing and melting processes. (a) Temperature-freezing time dependencies of DI water containing 0.05 (yellow line), 0.5 (pink line), and 5 ppm AuNPs (red line), and DI water (black line) in an ice bath of a salt-ice-water system (the temperature was controlled to ca. -20 °C). The green and blue lines represent environmental temperatures measured at the center point and at a corner point, respectively, in the ice bath. Before the experiments, sealed tubes with water samples were placed on a platform of an orbital shaker, operating at 150 rpm, under illumination with green LEDs for 4 h. (b) Temperature-melting time dependencies of frozen DI water containing 0.05 (yellow line), 0.5 (pink line), and 5 ppm AuNPs (red line), and frozen DI water (black line) in a cold-water bath (the temperature was controlled to ca. 5 °C). The blue line represents the environmental temperature measured at a corner point in the cold-water bath.


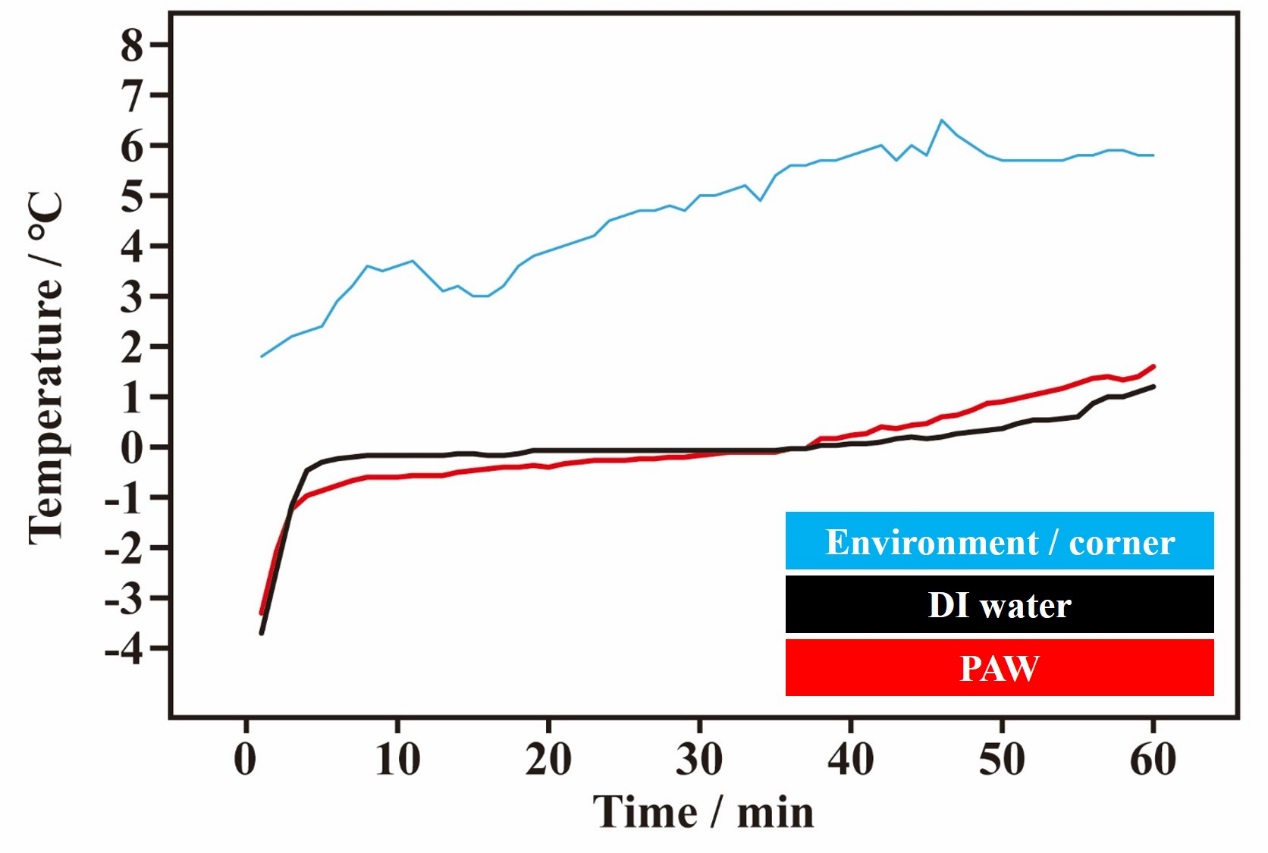


Figure S6. Reproducible experiments (Figure 4b in text) regarding temperature-time dependencies of plasmon-activated water (PAW) and deionized (DI) water (based on gold nanoparticle (AuNP)-coated filter paper and AuNP-free filter paper, respectively, under illumination with green LEDs in preparations) during melting processes after freezing of PAW and DI water. Temperature-melting time dependencies of frozen PAW (red line) and frozen DI water (black line) in a cold-water bath (the temperature was controlled to ca. 5 °C). The blue line represents the environmental temperature measured at a corner point in the cold-water bath.


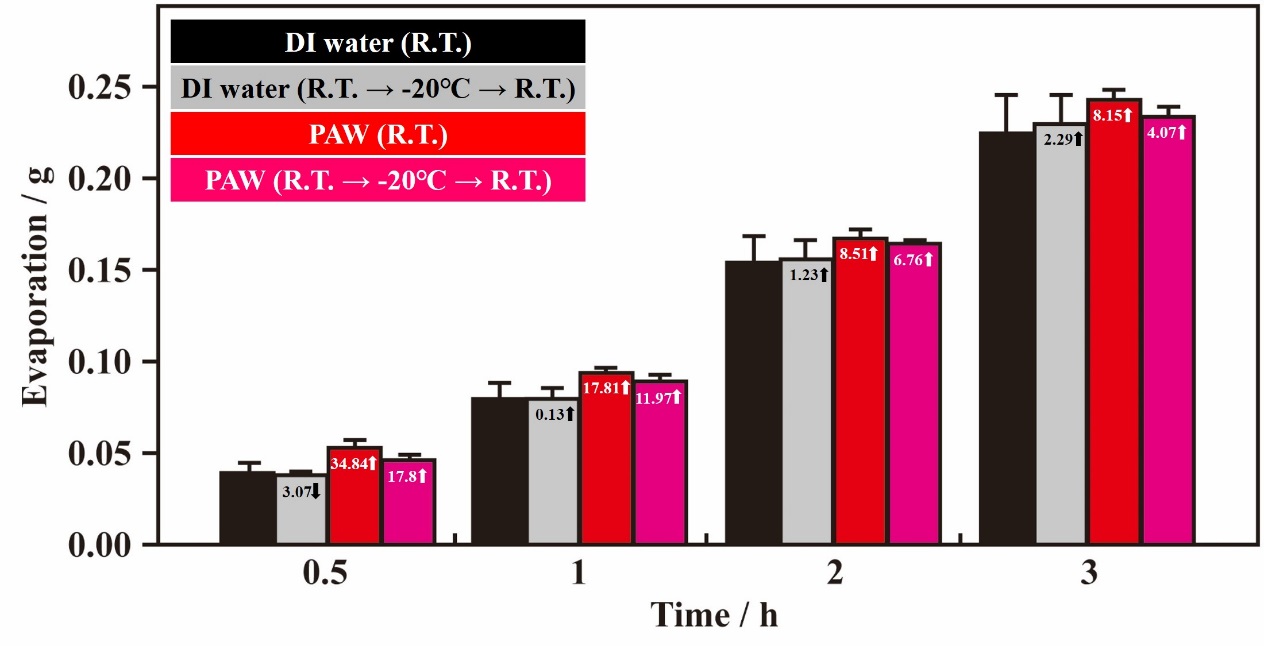


Figure S7. Evaporation quantities (g) with time of fresh plasmon-activated water (PAW) (red block) and deionized (DI) water (black block), and melted ice (PAW, pink block) and ice (DI water, gray block) from frozen PAW and DI water, respectively. The evaporation experiments were performed at 1 atm and room temperature.


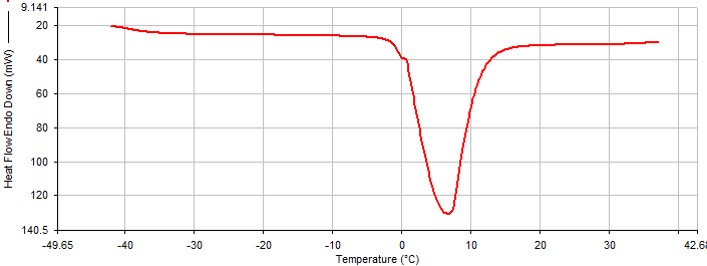


(a)


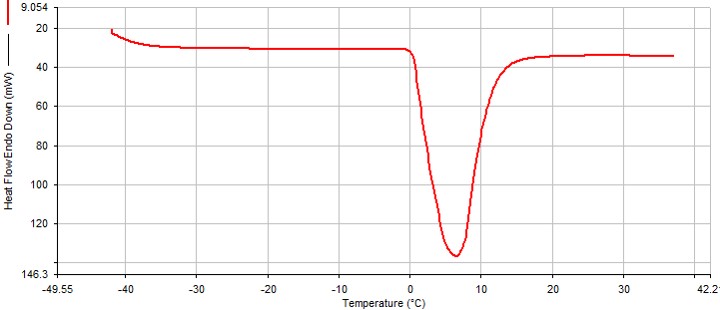


(b)


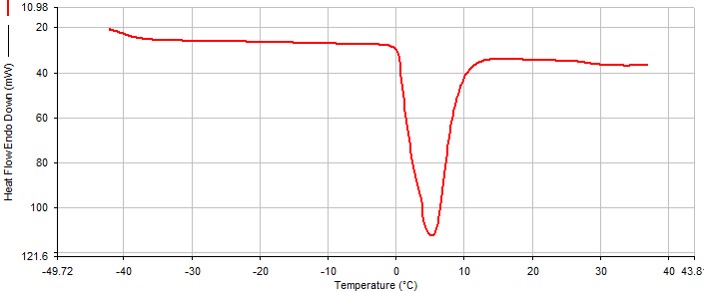


(c)


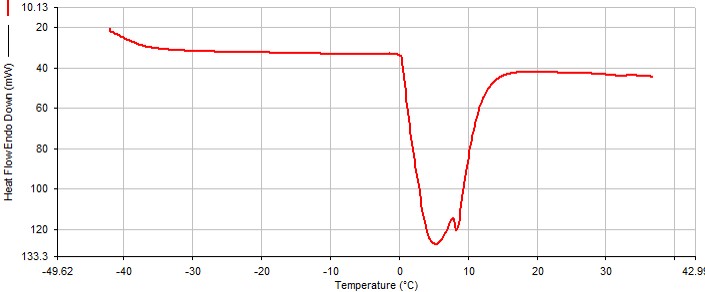


(d)


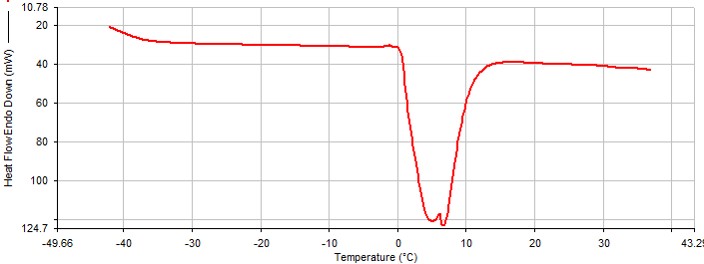


(e)


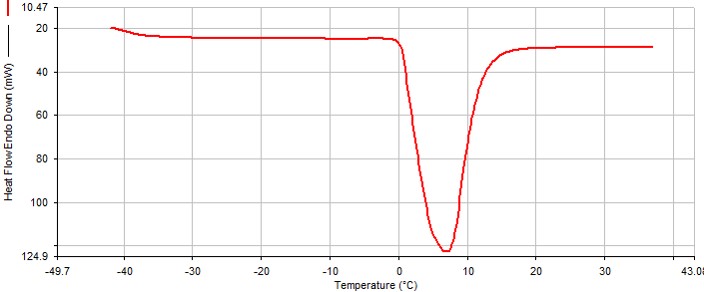


(f)


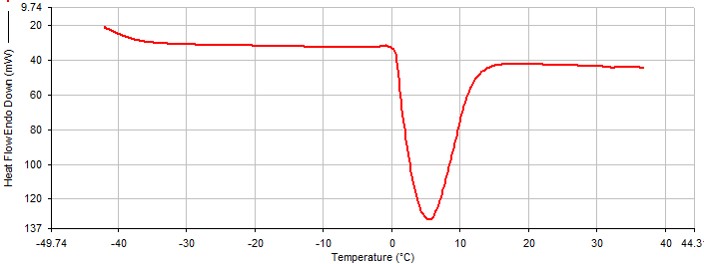


(g)


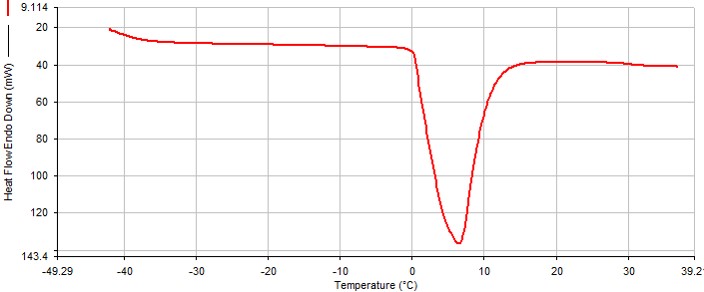


(h)


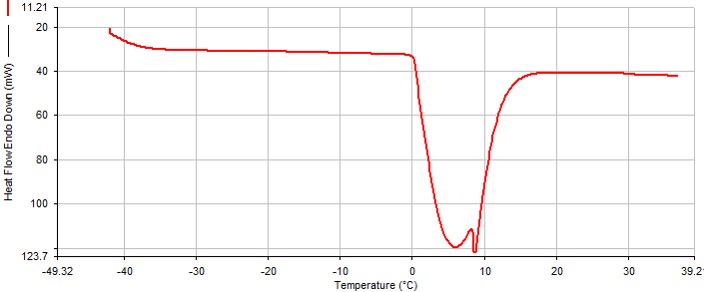


(i)


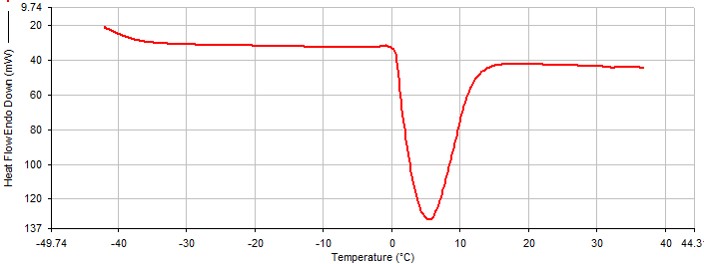


(j)

Figure S8. Reproducible experiments (Figure 7 in the text) of DSC thermodynamic responses of plasmon-activated water (PAW) and deionized (DI) water. The experiments were performed under the same heating rate of 10 °C min^−1^ from -40 to 40 °C. (a)~(e): DI water samples. (f)~(j): PAW samples.


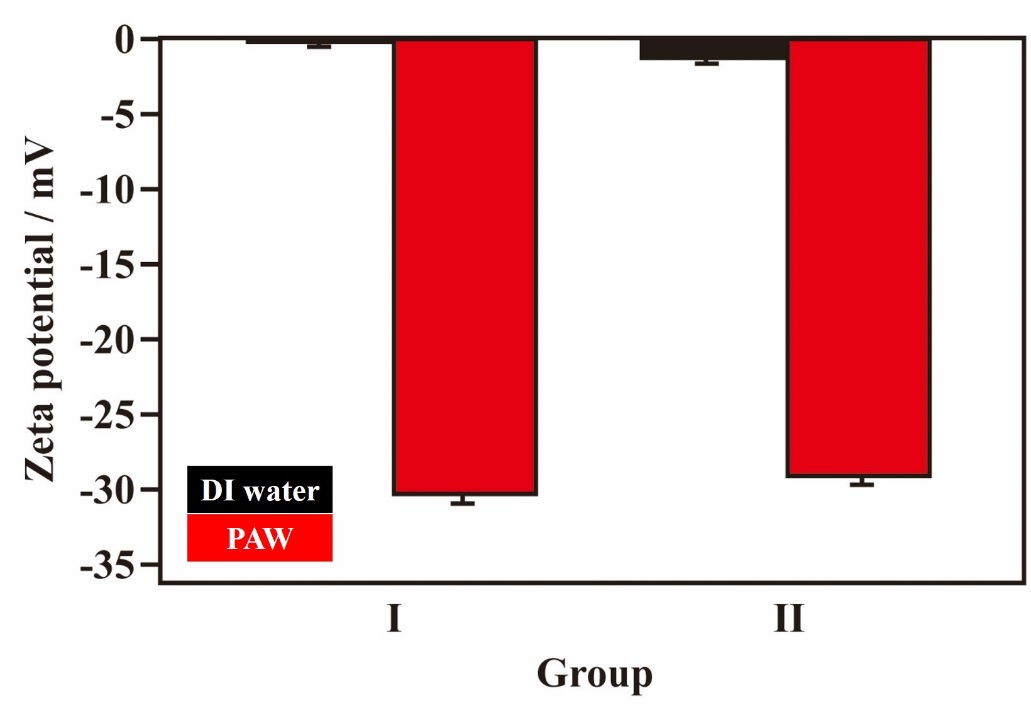


(a)


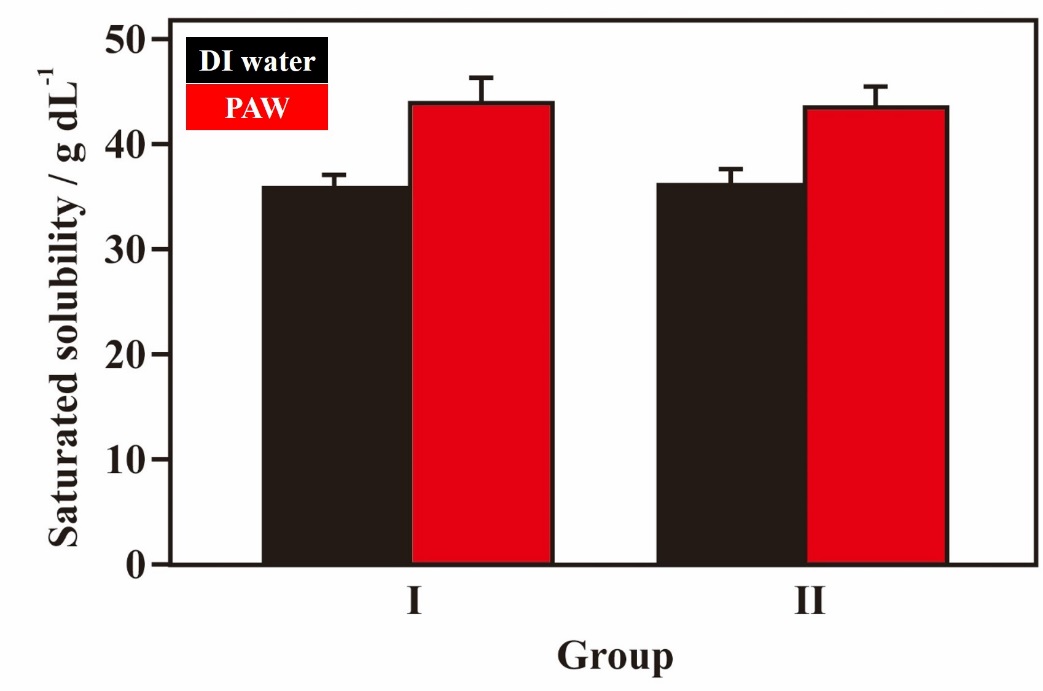


(b)

Figure S9. Zeta potentials of plasmon-activated water (PAW) and deionized (DI) water, and saturated solubilities of NaCl in them under ambient laboratory air. (a) Zeta potentials of PAW and DI water. (b) Saturated solubilities of NaCl in PAW and DI water. PAW and DI water in group I represent water samples discussed in Figure 1 of the text, while PAW and DI water in group II represent water samples discussed in Figure 4 of the text.
